# Wet Media Milling Preparation and Process Simulation of Nano-Ursolic Acid

**DOI:** 10.3390/pharmaceutics17101297

**Published:** 2025-10-03

**Authors:** Guang Li, Wenyu Yuan, Yu Ying, Yang Zhang

**Affiliations:** 1 School of Chemistry and Chemical Engineering, South China University of Technology, Guangzhou 510640, China; 2 Guangzhou Tinci Materials Technology Co., Ltd., No. 8, Kangda Road, Huangpu District, Guangzhou 510730, China

**Keywords:** ursolic acid, wet media milling, population balance model, nanosuspension

## Abstract

**Background/Objectives**: Pharmaceutical preparation technologies can enhance the bioavailability of poorly water-soluble drugs. Ursolic acid (UA) has been found to possess anti-cancer and hepatoprotective properties, demonstrating its potential as a therapeutic agent; however, its hydrophobicity and low solubility present challenges in the development of drug formulations. This study investigates the preparation of a nano-UA suspension by wet grinding, researches the influence of process parameters on particle size, and explores the rules of particle breakage and agglomeration by combining model fitting. **Methods**: Wet grinding experiments were conducted using a laboratory-scale grinding machine. The particle size distributions (PSDs) of UA suspensions under different grinding conditions were measured using a laser particle size analyzer. A single-factor experimental design was employed to optimize operational conditions. Model parameters for a population balance model considering both breakage and agglomeration were determined by an evolutionary algorithm optimization method. By measuring the degree to which UA inhibits the colorimetric reaction between salicylic acid and hydroxyl radicals, its antioxidant capacity in scavenging hydroxyl radicals was indirectly evaluated. **Results**: Wet grinding process conditions for nano-UA particles were established, yielding a UA suspension with a D50 particle size of 122 nm. The scavenging rate of the final grinding product was improved to three times higher than that of the UA raw material (D50 = 14.2 μm). **Conclusions**: Preparing nano-UA suspensions via wet grinding technology can significantly enhance their antioxidant properties. Model regression analysis of PSD data reveals that increasing the grinding mill’s stirring speed leads to more uniform particle size distribution, indicating that grinding speed (power) is a critical factor in producing nanosuspensions.

## 1. Introduction

Currently, over 40% of marketed drugs have poor solubility; many lead compounds during new drug discovery show poor solubility in aqueous media, hindering their pharmaceutical development [1,2]. Improving the apparent solubility and dissolution rate of an insoluble active pharmaceutical ingredient (API) using pharmaceutical preparation technology has become an effective strategy for these API candidates. Methods to improve drug solubility include modifying the molecular structure (e.g., developing salts or prodrugs, or complexing with cyclodextrins [3,4,5]), using colloidal drug delivery systems (e.g., microemulsions [6]), or optimizing the apparent size and morphology (e.g., reducing particle size or producing amorphous drugs [7,8]). Micro-pulverization technology reduces the apparent particle size of drug powders to between 1 and 10 μm. This increases the specific surface area of poorly soluble drugs, thereby enhancing their dissolution rate [9]. According to the Ostwald–Freundlich equation, the solubility improvement effect is significant when the particle size is less than 1–2 μm, especially under 200 nm [10,11]. Therefore, numerous poorly soluble drugs are marketed in the form of nanosuspensions.

Depending on the initial materials used, nanosuspension production methods can be categorized as top-down or bottom-up [12]. Wet media grinding is one of the top-down methods [13], achieving particle size reduction through collisions between drug particles and grinding media. Advantages of this method include low energy consumption and continuous batch operation, making it the preferred method for producing nanoformulations of poorly soluble drugs [14,15]. For example, Megestrol acetate^®^ of Par Pharmaceuticals and Morphine sulfate^®^ of Pfizer Pharmaceuticals are processed by wet media grinding drug particles to meet the requirements of nanomaterials [16].

The ursolic acid studied in this paper is a natural active compound with a pentacyclic triterpene structure that can be isolated and purified from various natural plants such as apples and rosemary [17]. It exhibits significant antitumor effects [18], anti-cancer effects [19], hepatoprotective effects [20], and other benefits [21,22]. However, ursolic acid has a hydrophobic structure with very low solubility in water, only 1.02 × 10^−4^ $\mathrm{mg}\cdot L^{-1}\;\left(25\;{}^{\circ}C\right)$ [23], which severely impairs its medicinal efficacy. In order to overcome the insolubility of ursolic acid, some preparation methods have been reported in previous studies, such as liposomes [24], the Nanofiber Process [25], nanocrystals [26], cyclodextrin encapsulation [27], etc. As a widely used preparation method in the industry, wet media milling can effectively improve solubility by reducing particle size, but no research on ursolic acid has been reported yet. This aim of this study is to prepare a nano-ursolic acid suspension by wet grinding, investigate the influence of process parameters on particle size, enhance their application performance, and explore the rules of particle breakage and agglomeration by combining model fitting.

## 2. Materials and Methods

### 2.1. Materials

**Figure 1:**
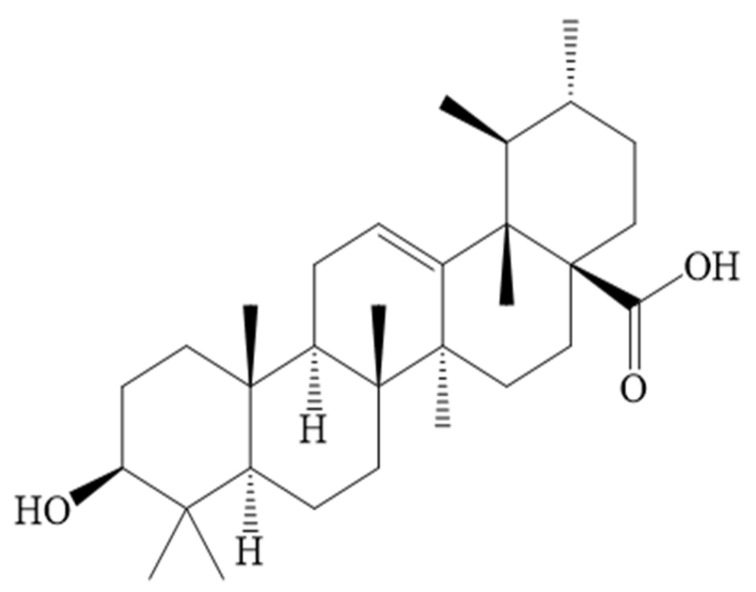
Chemical structure of UA.

Ursolic acid (the chemical formula is C_30_H_48_O_3_, with a molecular weight of 456.7. UA, 95%, purchased from Guilin Sanleng Biotechnology Co. (Guilin, China); molecular formula shown in Figure 1), sodium hex metaphosphate (SHMP, AR), sodium dodecyl benzene sulfonate (SDBS, CP), polyethylene glycol 6000 (PEG-6000, CP), hydroxyethyl cellulose (HEC, AR), hydroxypropyl methyl cellulose (HPMC, AR), Tween-20 (CP), polyvinyl pyrrolidone (PVP, AR), ferrous sulfate (AR), hydrogen peroxide solution (30%AR), salicylic acid (AR), absolute ethanol (AR). SHMP, Tween-20, and salicylic acid were purchased from Tianjin Kemiou Chemical Reagent Co., Ltd. (Tianjin, China); HEC, HPMC, and PVP were purchased from Shanghai Aladdin Biochemical Technology Co., Ltd. (Shanghai, China); ferrous sulfate, hydrogen peroxide solution, and absolute ethanol were purchased from Guangzhou Chemical Reagent Factory (Guangzhou, China); SDBS and PEG-6000 were purchased from Shanghai Peakinfo Chemical Reagent Co., Ltd. (Shanghai, China) and Sinopharm Chemical Reagent Co., Ltd. (Shanghai, China), respectively. Deionized water used is self-made in the laboratory.

### 2.2. Method

#### 2.2.1. Wet Media Milling

**Figure 2:**
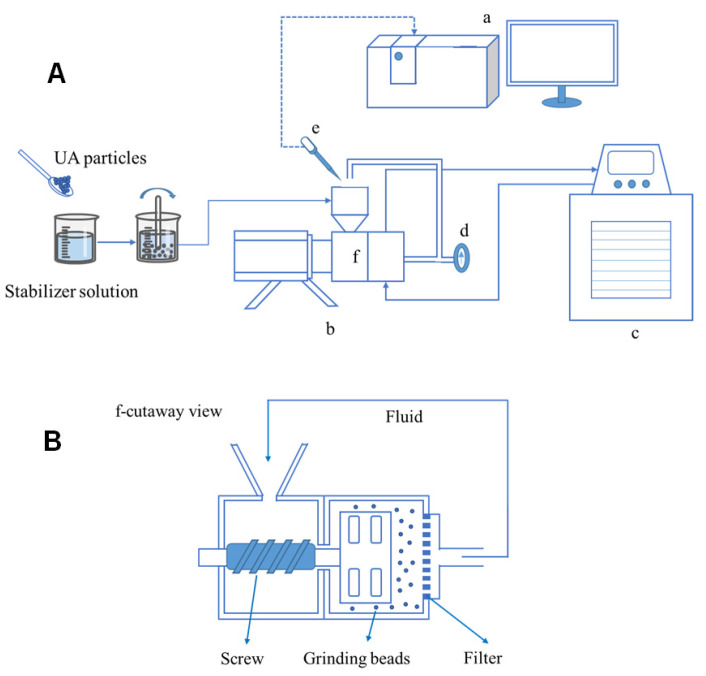
(**A**): Wet grinding process flow chart: (a) laser particle size analyzer; (b) laboratory-scale grinding machine; (c) water bath; (d) temperature indicator; (e) pipette; (f) grinding chamber. (**B**): Cross-section of wet grinding.

Before the grinding experiments began, the stabilizer was weighed with a precision balance (Mettler Toledo AL 204, Greifensee, Switzerland) into a clean beaker, after which it was added to deionized water under stirring (see Figure 2). Then, UA was added into the stabilizer solution, stirred until well dispersed. Media milling experiments were performed using a laboratory-scale grinding machine (WAB DYNO^®^, Basel, Switzerland), with yttrium-stabilized zirconium oxide grinding beads (with a nominal diameter of 0.1 mm and 0.3 mm) being used. The suspension was driven by the screw to the grinding chamber and finally returned to the hopper through a pipe with a temperature indicator. A constant-temperature water bath set to 273 K was used to cool the grinding chamber and the temperature was monitored to ensure it did not exceed 303 K. To avoid clogging, a low mass fraction of UA (2.0% *w*/*v*) was used throughout the grinding experiments. During the milling process, samples of the suspension were taken at regular intervals from the hopper for size analysis.

#### 2.2.2. Particle Size Measurement

The UA suspension was sampled during the milling process, analyzed by laser particle size analyzer (Malvern Panalytical Mastersizer 3000, Malvern, UK), and the cumulative particle size distribution D50 of the measurement results was used to represent the particle size.

#### 2.2.3. Scanning Electron Microscopy (SEM)

For solid-phase characterization, a part of the milling suspension was centrifuged at 2000 rpm for 20 min (TDL-80-2B, China), and then the solids obtained by centrifugation were dried at 60 °C for 12 h. An ultrahigh-resolution field emission scanning electron microscope (Hitachi Su8220, Tokyo, Japan) was used to observe the surface morphology of UA raw materials and grinding products. All samples were sputtered with a thin film of gold before testing. The measurements were carried out using an acceleration voltage of 5 kV.

#### 2.2.4. X-Ray Powder Diffraction (XRD)

For the obtained UA grinding suspension, after sampling, centrifugation, and drying, the sample was tested by Malvern Panalytical X-ray powder diffractometer (Panalytical X’ pert Powder, Almelo, Holland). The experiments were carried out at the 3 KW Cu Ka radiation with a Pixel 1D array detector. The scanning angle was between 5° and 60° with a step width of 0.013°.

#### 2.2.5. Differential Scanning Calorimetry (DSC)

A TA DSC (DSC 25, New Castle, DE, USA) was used to determine the simples at equilibrium temperature of 25 °C, terminal temperature of 310 °C, with a heating rate of 10 °C/min. Nitrogen purge gas flow pressure was set to 0.1 MPa.

#### 2.2.6. Oxidation Resistance Test

The antioxidant properties of UA have been reported, and the experiment was designed to characterization [28]. Specifically, at first, the salicylic acid ethanol solution reacts with hydroxyl radical (generated by hydrogen peroxide solution and ferrous ion) to generate a chromogenic substance. The concentration of the chromogenic substance can be detected by ultraviolet spectrophotometer (Shimadzu Uvmini-1240, Suzhou, China), and it shows strong absorption near 510 nm. After adding UA, some hydroxyl radicals can be eliminated, so the content of chromogenic substance in the solution will also decrease. In order to investigate the influence of particle size on the reaction activity of UA, the reaction between UA and hydroxyl radical was used to indirectly test the activity of UA during the milling process. A 1 mL quantity of uniformly dispersed UA suspension was taken from the system with a pipette when the grinding time was 0 min, 10 min, 30 min, and 60 min, respectively, and put into a clean beaker with 9 mL of water for dilution. Then, 1 mL of 10 mmol/L${\;\mathrm{FeSO}}_{4}$ solution, 1 mL of 30 mmol/L $H_{2}O_{2}$ solution, and 1 mL of 30 mmol/L salicylic acid ethanol solution were added into beaker and stirred well. In order to avoid the influence of UA particles on the detection of the ultraviolet spectrophotometer, the reaction solution was centrifuged at 2000 rpm for 10 min (TDL-80-2B, Shanghai, China); the centrifuged solution was transferred into a 1 cm quartz cuvette afterwards, and the absorbance was scanned at its maximum absorption wavelength. The absorbance difference between blank solution and that added with UA can reflect the scavenging effect of UA on hydroxyl radicals. The scavenging rate is defined by the following formula:

(1) $$E\left(\%\right)=\frac{A_{0}-A_{x}}{A_{0}}*100\%$$

where $A_{0}$ represents the absorbance value of the blank solution; $A_{x}$ is the absorbance value of the reaction system after adding UA suspension.

#### 2.2.7. Model Fitting

In the process of wet milling, the breakage and agglomeration of material particles in the grinding chamber are involved; however, this process is difficult to observe and measure in situ in an enclosed grinding chamber. The population balance model was developed to describe the evolution process of particle size distribution in the process of particle change, including nucleation, growth, agglomeration, breakage, etc. It has been successfully applied to describe the particle behavior for many particle processing processes, such as crystallization, granulation, spray drying, and liquid–liquid extraction [29]. According to the actual situation, it has a variety of expressions. For the wet milling system in this paper, it mainly involves particle breakage and agglomeration processes [30]; the population balance model can be expressed as

(2) $$\begin{array}{cc} \frac{\partial n\left(v;t\right)}{\partial t} & =\underset{\left(1\right)}{\underbrace{\frac{1}{2}\int_{0}^{v}\beta \left(v-\epsilon ,\epsilon \right)n\left(v-\epsilon ;t\right)n\left(\epsilon ;t\right)d\epsilon }}-\underset{\left(2\right)}{\underbrace{n\left(v;t\right)\int_{0}^{\infty }\beta \left(v,\epsilon \right)n\left(\epsilon ;t\right)d\epsilon }}+\\ & \underset{\left(3\right)}{\underbrace{\int_{v}^{\infty }a\left(\epsilon \right)b\left(v|\epsilon \right)n\left(\epsilon ;t\right)d\epsilon }}-\underset{\left(4\right)}{\underbrace{a\left(v\right)n\left(v;t\right)}} \end{array}$$

On the left side of Equation (2), $n\left(v;t\right)$ is the particle number density function: if there are dN particles in the suspension per unit volume in the system, and the particle size range is v to v+dv, then the density function at this size and time is n=dN/dv. On the right side of the same equation: (1) the generation rate of particles of volume *v* due to the aggregation of smaller particles; (2) the disappearance rate of particles of volume *v* due to aggregation with other particles; (3) the rate at which particles of volume *v* are produced due to the breakage of larger particles; (4) the rate at which particles of volume *v* disappear due to crushing, where $\beta \left(v,\epsilon \right)$ is the agglomeration rate kernel based on volume, which describes particles of volume *v* and ϵ colliding to form particles of volume v+ϵ. $a\left(v\right)$ is the breakage rate kernel, describing the breaking frequency of particles of volume v;$\;b\left(v|\epsilon \right)$ is the breakage distribution function.

In a study of population balance models, various rate kernel expressions were developed by researchers to apply to different material systems [31]. For the model fitting of this experiment, the breaking rate kernel is represented by a power-law rate kernel:

(3) $$a\left(v\right)=K_{1}v^{\mu }$$

where $K_{1}$ is the breakage frequency constant, *v* is the particle volume, and μ is the power factor. Using different agglomeration rate kernels to fit the experimental data, it is found that the diffusion-limited rate kernels have the best fitting effect. The diffusion-limited rate kernel is given by

(4) $$\beta \left(v_{1},v_{2}\right)=K_{2}\left(v_{1}^{\frac{1}{y_{1}}}+v_{2}^{\frac{1}{y_{2}}}\right)\left(v_{1}^{-\frac{1}{y_{1}}}+v_{2}^{-\frac{1}{y_{2}}}\right)$$

where $K_{2}$ is the agglomeration frequency constant, $v_{1},v_{2}$ are the different particle volume, and $y_{1},y_{2}$ are the power factor.

$b\left(v|\epsilon \right)$ stand for the particle distribution of parent volume of ε during particle crushing [32], expressed by the following formula:

(5) $$b\left(v|\epsilon \right)=\frac{pv^{c}{\left(\epsilon -v\right)}^{c+\left(c+1\right)\left(p-2\right)}\Gamma \left(c+\left(c+1\right)\left(p-1\right)+1\right)}{\epsilon^{p\cdot c+p-1}\Gamma \left(c+1\right)\Gamma \left(c+\left(c+1\right)\left(p-2\right)+1\right)}$$

where Γ is the gamma function, p is the number of broken particles, and c is the parameter that determines the shape of the distribution function.

Using the discretized population balance method, the partial differential equation is transformed into a set of ordinary differential equations. In each volume interval $\left[v_{i},v_{i+1}\right)$, the number concentration is defined:

(6) $$N_{i}\left(t\right)=\int_{v_{i}}^{v_{i+1}}n\left(v;t\right)dv$$

And the population balance model is changed to

(7) $$\;\frac{dN_{i}}{dt}=\frac{1}{2}\overset{i-1}{\underset{j=1}{\sum }}\beta \left(i-j,j\right)N_{i-j}N_{j}-N_{i}\overset{\infty }{\underset{j=1}{\sum }}\beta \left(i,j\right)N_{j}+\overset{\infty }{\underset{j=i+1}{\sum }}a\left(j\right)b\left(i|j\right)N_{i}-a\left(i\right)N_{i}$$

The parameters of breakage and agglomeration in the above formula are unknown, so parameter estimation should be carried out in combination with the particle distribution data measured in experiments. A graphical software package (Population Balance Parameters Estimation version 1.0) developed by our research group was used to calculate the relevant parameters of the core of the breakage and agglomeration rate; it can predict the particle size distribution at different time points in the grinding process. Wynn’s discrete equilibrium equation [33] numerically solves the population balance model to calculate the agglomeration term; Kumar and Ramakrishna’s method [34] is used to calculate the breakage term. The second-order backward differentiation formula is used to solve the set of ordinary differential equations. The evolutionary algorithm optimization method is used to determine the best parameters.

## 3. Results and Discussion

### 3.1. Media Milling Experiment

#### 3.1.1. Selection of Stabilizer

Due to its large specific surface area and high surface energy, nanoparticles will tend to aggregate spontaneously. Hence, it is necessary to add substances called stabilizers to prevent the spontaneous aggregation of the nanoparticle system. Based on their mechanisms of action, stabilizers can be categorized into two types: ionic surfactants (e.g., SHMP, SDBS, PEG-6000, HEC, HPMC) and nonionic surfactants (e.g., PVP, Tween-20). Ionic surfactants can ionize in aqueous solutions, releasing charged ions that adsorb onto particle surfaces to form charge layers. The resulting electrostatic repulsion between particles effectively prevents agglomeration. They exhibit high stability and remarkable efficacy, though they are sensitive to electrolytes and pH levels. Nonionic surfactants, on the other hand, significantly reduce interfacial tension through steric hindrance, ensuring uniform distribution of nanoparticles within the dispersion medium. Their key advantage lies in their independence from electrostatic repulsion, enabling functionality in electrolyte systems, but they are relatively sensitive to temperature.

**Figure 3:**
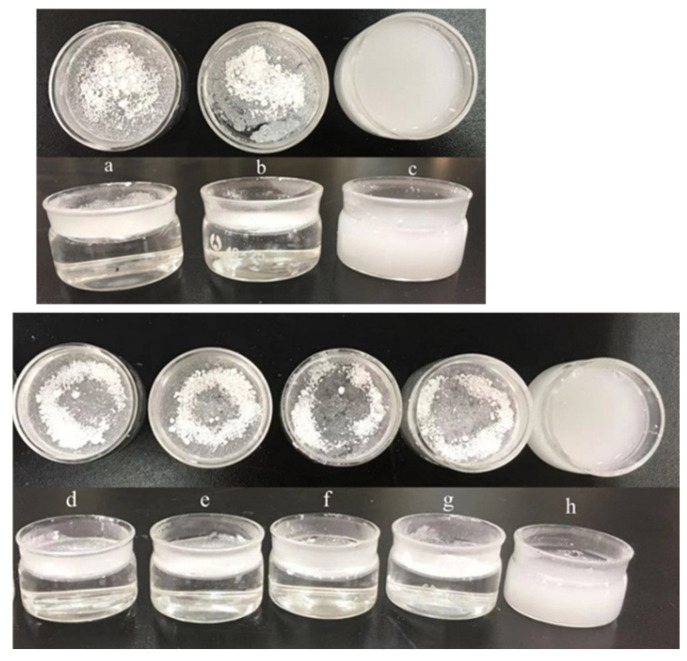
The different stabilizer screening experiments: (a–h) are water, SHMP, SDBS, PEG-6000, PVP, HEC, HPMC solution (mass fraction of 1%), and Tween-20 solution (volume fraction of 5%) in turn.

In the pre-experiment, it was found that UA would float on the liquid surface in the aqueous medium, which is not conducive to flowing with the liquid when milling. Therefore, a good stabilizer should interact with UA to make it suspend in the system, which is convenient for subsequent milling experiments. The simplified evaluation method was used to preliminary screening of stabilizers. The specific operation was to prepare a certain concentration of stabilizer solution and add a certain amount of UA to observe the solution state. Figure 3 shows the result of the screening experiments.

It can be observed from Figure 3 that under the condition of SDBS and Tween-20 as stabilizers, UA could be dispersed in solution to form suspension, while under the condition of other stabilizers, UA floated on the surface of the solution. Therefore, SDBS and Tween-20 were initially selected for subsequent experiments.

**Figure 4:**
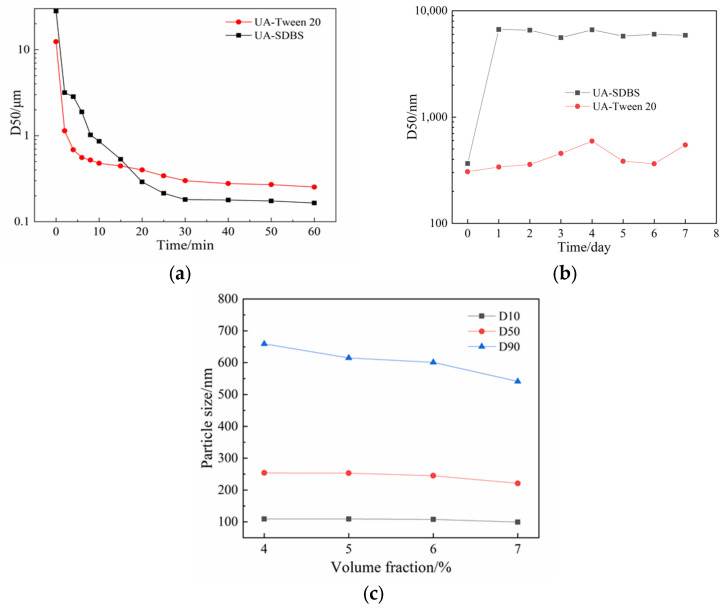
Results of D50 particle size: (**a**) variation over milling time for suspensions prepared under two stabilizer conditions: SDBS and Tween-20; (**b**) variation over storage time for suspensions prepared under two stabilizer conditions: SDBS and Tween-20; (**c**) variation with different Tween-20 concentrations.

Prior to the grinding experiments, the Tween-20 solution (volume fraction of 4%) and the SDBS solution (mass fraction of 1%) were prepared, respectively, and UA (mass fraction of 2%) was added to form suspension by stirring evenly. Under experimental conditions—0.3 mm grinding beads, rotating speed of 3000 rpm, grinding time set to 60 min—samples were taken at regular intervals for particle size analysis. The results are shown in Figure 4.

As can be seen, the particle size is smaller when SDBS is used as the stabilizer. The D50 particle size of the product obtained was 165 nm, whereas the D50 particle size of the Tween-20 grinding product was 265 nm. To further investigate the stability of the UA suspension with the two stabilizers, the grinding products were placed at ambient temperature (298 K) and sampled regularly every day to measure the particle size and check for agglomeration of particles in the system over a week. Figure 4b shows the variation in particle size as a function of storage time (days). The results show that Tween-20 has a stabilizing effect on the UA suspension system. The particle size fluctuated in the range of 400–600 nm during a week, increasing slightly compared to the initial stage. In contrast, the particle size of the product of SDBS was small during the milling process, but the D50 measured of stationary suspension was 366 nm when the product was collected, and it increased to 6.68 μm after storage for one day. Meanwhile, after the sample had been resting for a week, it was found that the suspension containing SDBS had particles deposited at the bottom of the beaker, but the grinding product with Tween-20 still maintained the suspension state. According to the reported stability study of nanosuspension formed by stabilizer and drug composition [35], the difference in stability between SDBS and Tween-20 is speculated to be the difference in HLB value. As the HLB value of Tween-20 (HLB = 16.7) is greater than that of SDBS (HLB = 10.6), UA particles with Tween-20 attached to the surface can be well dispersed in the aqueous medium. For hydrophobic UA particles, the surfactants with high HLB values are preferred. Based on the above experiments, the insufficient stabilization effect of SDBS was obvious, and the subsequent experiments were carried out with Tween-20 as the stabilizer.

It can be observed from Figure 4a that the number of material particles broken in the initial stage increases and the particle size gradually decreases as milling time increases. For small particles, due to their spontaneous aggregation, the particle size in the suspension system finally reaches equilibrium state, after which the extension of process time does not help in reducing particle size but causes energy loss due to long-time operation. Therefore, it is necessary to find the appropriate milling time as an experimental condition. The experiment was carried out under the condition of Tween-20 as the stabilizer (volume fraction of 4%) and 3 h for milling time. A laser particle size analyzer was used to monitor the change in particle size over time. Cumulative particle sizes D10, D50, and D90 were used to indicate the change in particle size over the process time (see Figure 4c). It was observed that the particle size of UA had not changed after 60 min of grinding, indicating the particle size maintained a small fluctuation within a certain range, which proved that the breakage and agglomeration of substances were in equilibrium at this time; therefore, the milling time of subsequent experiments was set to 60 min to ensure the suspension system reached an equilibrium state.

Generally, a low stabilizer concentration cannot inhibit the aggregation of nanoparticles, while a high concentration may also promote Ostwald ripening, resulting in an increase in particle size [35]. This study investigated the influence of different stabilizer concentrations (volume fraction of 4%, 5%, 6%, and 7%) on grinding efficiency. The rotating speed of the grinder shaft was set to 3000 rpm, and the process time was 60 min. Figure 4c shows the particle size influenced by the concentrations of Tween-20.

It was found that increasing the stabilizer concentration narrowed the particle size distribution in the system and decreased the proportion of large particles, which is conducive to obtaining products with a relatively uniform particle size. However, during the experiment, the return flow rate decreased significantly with an increase in stabilizer concentration, even at the same rotating speed, due to an increase in suspension viscosity. When testing the Tween-20 solution at a volume fraction of 10%, it was difficult for the suspension to reflow into the grinding chamber, as the stabilizer Tween-20 is an oily liquid whose concentration would affect the viscosity and other properties of the suspension system, as well as the grinding efficiency. The higher the viscosity, the more the movement of the grinding medium is dampened by the fluid, thus reducing the energy available for size reduction. Therefore, in the experimental process, the relationship between stabilizer concentration and target particle size should be considered, and a medium stabilizer concentration should be used for grinding experiments. Therefore, the optimal concentration of stabilizer in our experiment is 5–6%.

#### 3.1.2. Effect of Grinding Speed and Grinding Beads Size

**Figure 5:**
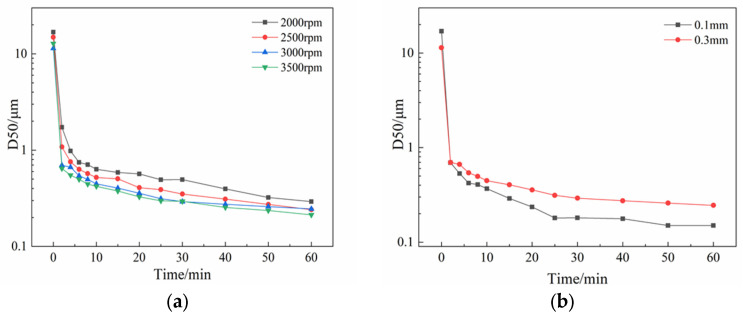
Results of D50 particle size variation over milling time in suspensions under (**a**) different grinding speeds; (**b**) two different grinding bead sizes.

The horizontal wet grinding machine used in the laboratory relies on the high-speed rotation of the main shaft to fully mix the grinding medium and material particles and increase the breakage frequency of material particles [36]. Hence, the grinding speed has an important impact on the minimum particle size and particle size distribution of the final product. Tween-20 solution was prepared with a volume fraction of 6%, added to UA with a mass fraction of 2% to form suspension, using 0.3 mm grinding beads to conduct experiments at speeds of 2000 rpm, 2500 rpm, 3000 rpm, and 3500 rpm, respectively. The process time was 60 min. The results are shown in Figure 5.

For the preparation of nanosuspension by wet media milling, particle breakage is the main mechanism, compared to aggregation in the grinding process. Therefore, the cumulative particle size D50 monotonously reduces to the grinding limiting size over time. In order to describe the relationship between D50 and time [37], the kinetic model expression of particles breakage is as follows:

(8) $$D50\left(t\right)=D_{lim}+{\left[{\left(D50\left(0\right)-D_{lim}\right)}^{1-n}-\left(1-n\right)*k*t\right]}^{\frac{1}{1-n}}$$

where $D_{lim}$ represents the grinding limit size, which is larger than the final size in the experiment; *k* is the crushing rate constant; *n* is the kinetics order. $D_{lim}$, *k*, and *n* were estimated with the logarithm of the experimental data by the Marquardt–Levenberg algorithm by using 1stOpt software (Professional Version 1.5). Statistical analysis of the parameters was performed and the results are shown in Table 1.

As can be seen, the nth-order kinetics model has good fitting capability. From the fitting results, in general, with the increase in rotating speed, the particle breakage rate k increases, and the decrease in grinding limiting size $D_{lim}$ is consistent with the experimental data.

Based on the above experimental results, the optimal experimental conditions of UA wet milling are as follows: grinding speed set to 3500 rpm, Tween-20 at 6% (volume fraction) as stabilizer, and a process time of 60 min. In this study, the effect of grinding bead size on the size of the wet milling product was also investigated. In general, the smaller the grinding beads, the greater the collision frequency between material particles and beads, resulting in a small particle size [34]. Under the above operating conditions, two sizes of grinding beads of 0.1 mm and 0.3 mm were investigated in this study, and the particle sizes of the UA suspension at the set time points were monitored.

Figure 5b shows the result of UA D50 particle size in the obtained experimental suspension with different sizes of grinding beads. It can be seen that the experimental results are consistent with the assumption that the product obtained using 0.1 mm grinding beads has a smaller particle size. However, it was observed that using grinding beads of 0.1 mm would cause the grinding chamber to heat up considerably. This indicates that with smaller grinding media, the kinetic energy of the grinding beads is transferred to the material particles, causing breakage. It also suggests that an unknown amount of energy is dissipated into heat [38]. Therefore, it is essential to control the grinding speed and viscosity of the grinding media during the wet grinding process to prevent the grinding chamber temperature from soaring and causing degradation of the product.

### 3.2. Product Characterization

#### 3.2.1. SEM

**Figure 6:**
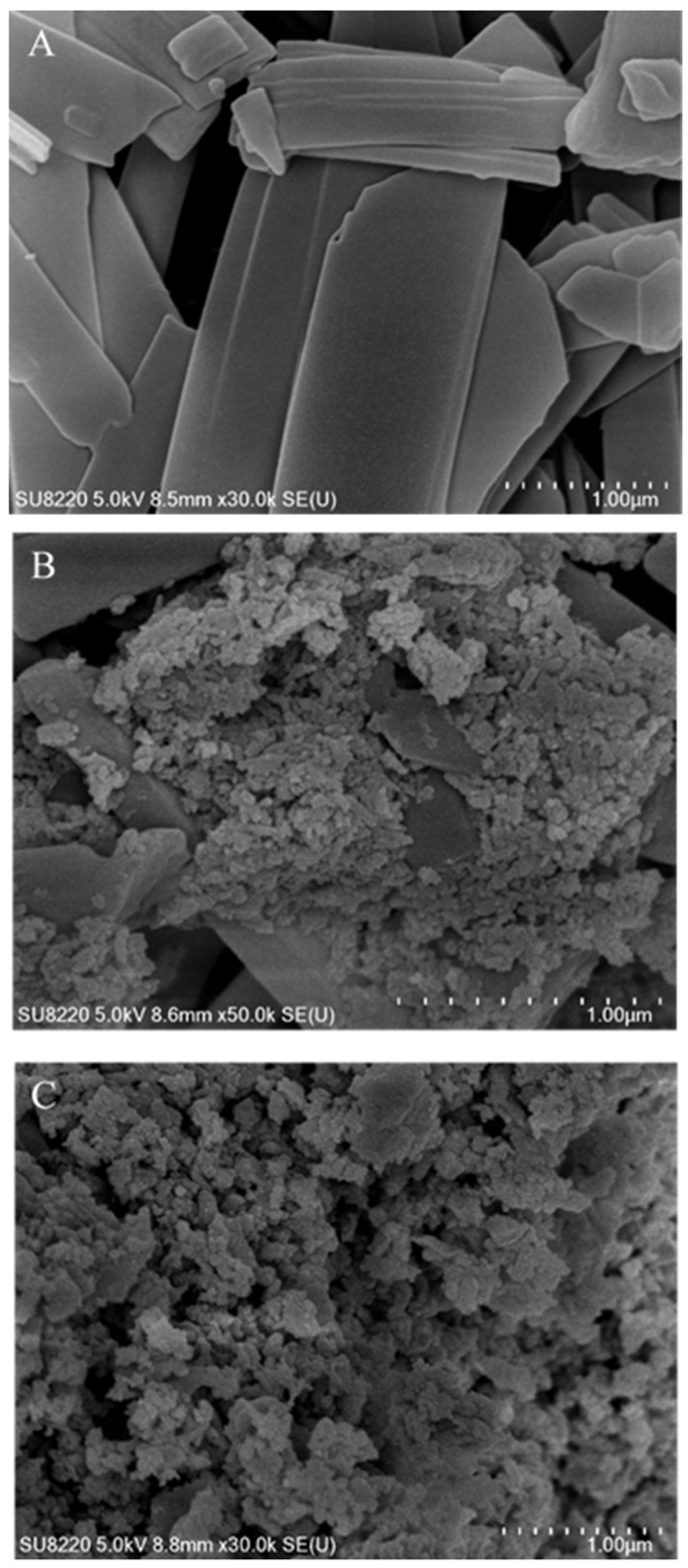
SEM microscopic analysis of UA raw materials (**A**) and of grinding products with SDBS (**B**) and Tween-20 (**C**) as stabilizers.

The crystal surface morphology can be clearly and intuitively observed by using the SEM, so as to compare the changes in the morphology of the UA raw materials and grinding products of different stabilizers. Because the product obtained by wet milling was suspension, some samples were taken and dried into powder and observed under an SEM after centrifugation. As can be seen from the SEM pictures (see Figure 6), the micro-structure of UA raw materials was a thin, lamellar solid. After wet milling, clastic solids were obtained, which agglomerated due to the way the sample was prepared, which can be observed in the SEM pictures of grinding products under different stabilizer conditions. An obvious difference was observed in the milling products with SDBS, which exhibited a lamellar crystal structure consistent with that of the UA raw material. This phenomenon was not observed in products with Tween-20 as stabilizer. In combination with the stability inspection experiments shown in Figure 5, it is speculated that the inadequate stabilization effect of SDBS on the UA particles caused the colloidal solids to dissolve and combine to form the lamellar structure, i.e., the Ostwald ripening phenomenon. Comparative experiments demonstrate that Tween-20, a polymer with a long-chain branched structure, can effectively disperse highly hydrophobic UA nanoparticles and maintain long-term stability through steric hindrance effects. Even after solid–liquid separation operations such as centrifugation or drying, it prevents particle growth, thereby preserving the stability of particle morphology and size distribution.

#### 3.2.2. XRD

**Figure 7:**
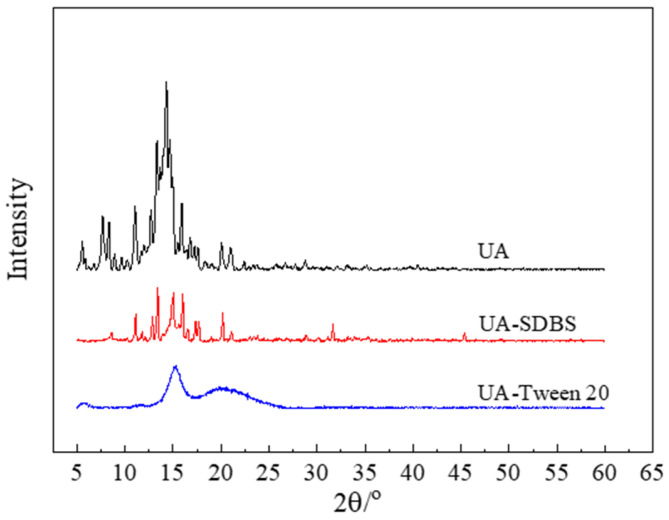
XRD analysis of UA raw materials and grinding products with SDBS and Tween-20.

The influence of the milling process and stabilizer addition on the crystal structure was investigated by subjecting UA raw material and centrifugally prepared grinding sample powder to XRD, as shown in Figure 7. As shown, the diffraction pattern that the characteristic peaks of UA raw materials are 13.998°, 14.352°, and 15.942°. In comparison, the characteristic peaks of the grinding products with SDBS are 13.421°, 15.074°, and 16.057°. These peaks shift and the diffraction intensity is low, indicating that the crystal structure is damaged after milling. However, the grinding product with Tween-20 has a gentle diffraction pattern. Combined with the analysis results of the SEM, the broadening in the diffraction pattern can be attributed to the small particle size [39].

Unlike the spherical UA nanoparticles studied by Hu et al. [26], the UA nanosuspension prepared in this study exhibits an irregular granular shape, and the prepared UA particles using wet milling are smaller in size and retain a certain crystalline structure, as shown in Figure 7, not completely transforming into an amorphous state. The irregular granular shape is the result of high-frequency collisions between grinding beads and particles, and these collisions fragmented the particles into nanoscale sizes; therefore, the shape changed. Furthermore, although surface damage partially disrupted the crystal lattice, the original crystalline structure was partially preserved. These results indicated potential superior stability compared to previous studies. Compared to the bottom-up approach used for preparation of nano-UA in the literature [26]—which requires crystallization, high-speed stirring, and organic solvents—this study introduces a novel wet grinding method for preparing nano-UA suspensions that is simpler and easier to industrialize and uses water as media.

#### 3.2.3. DSC

**Figure 8:**
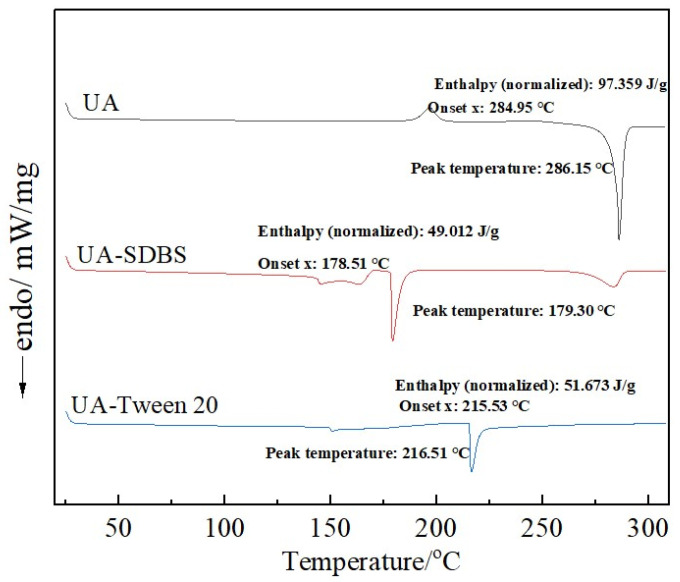
DSC analysis of UA raw materials and grinding products with SDBS and Tween-20.

A TA DSC 25 was used to analyze UA raw materials and grinding sample powders (see Figure 8). As shown, the DSC curve of the raw UA material shows a sharp endothermic event at 286.15 °C, which corresponds to the melting peak. However, the DSC curve of the grinding products differs greatly from that of the raw UA materials. The grinding products with SDBS as stabilizer exhibit an initial endothermic peak at 179.3 °C, followed by a second endothermic peak at 283.59 °C. Furthermore, the prepared grinding products with Tween-20 as stabilizer exhibit a single endothermic peak at 216.51 °C. Combined with the analysis results of particle size and SEM, the particle size of the grinding product with SDBS as stabilizer is smaller than that with Tween-20, resulting in a lower crystal melting temperature [40]. Because SDBS as stabilizer has a poor stability effect on the system particles, the particles aggregate and exhibit a similar lamellar structure to the raw materials, leading to the second endothermic peak.

#### 3.2.4. Results of Oxidation Resistance Test

The UA suspension with a cumulative particle size of 122 nm (D50) was successfully prepared under the optimum technological conditions: the grinding speed was 3500 rpm, the stabilizer was Tween-20 at 6% (volume fraction), and the grinding time was 60 min with 0.1 mm grinding beads. Then, 1 mL of 10 mmol/L${\;\mathrm{FeSO}}_{4}$ solution, 1 mL of 30 mmol/L $H_{2}O_{2}$ solution, and 1 mL of 30 mmol/L salicylic acid ethanol solution were added into the beaker while stirring well. To further evaluate the impact of particle size on the application performance of UA, a certain volume of the suspension was taken at a fixed time intervals under the optimal wet grinding conditions and transferred to a beaker. Then, 1 mL of 10 mmol/L${\;\mathrm{FeSO}}_{4}$ solution, 1 mL of 30 mmol/L $H_{2}O_{2}$ solution, and 1 mL of 30 mmol/L salicylic acid ethanol solution were added. The ultraviolet absorption spectra of the suspensions after centrifugation were measured to evaluate the anti-free-radical reactivity of UA suspensions with different particle sizes by calculating the scavenging rate. The evaluation results are shown in Table 2.

It can be observed that the free radical scavenging capacity of the UA nanosuspension is greatly improved when the particle size of the UA suspension is reduced from micrometer level to nanometer level. The scavenging rate of the final grinding product was improved up to three times higher than that of the UA raw material (D50 = 14.2 μm), indicating that the performance of the UA nanosuspension was superior to that of the UA microsuspension.

The results show that particle size would significantly impact UA reaction activity with salicylic acid and hydroxyl radicals, and it was hypothesized that UA reaction activity relies on the number of exposed surface groups, which would increase when UA particle size becomes smaller.

### 3.3. Results of Model Fitting

The population balance model was used to simulate the revolution of UA particles during wet milling and to investigate the effect of process conditions, such as grinding speed, on particle size variation. The fitting parameters for the breakage rate kernel and agglomeration kernel are shown in Table 3.

**Figure 9:**
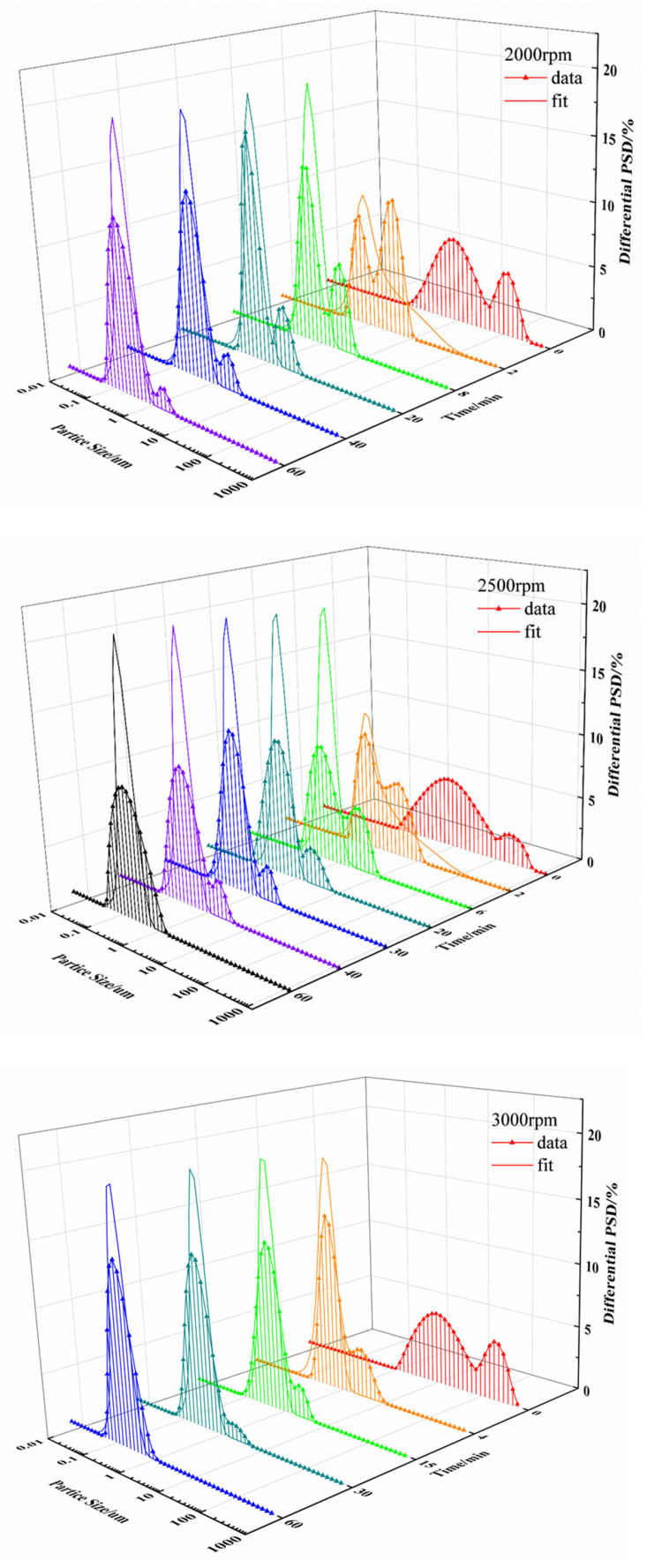

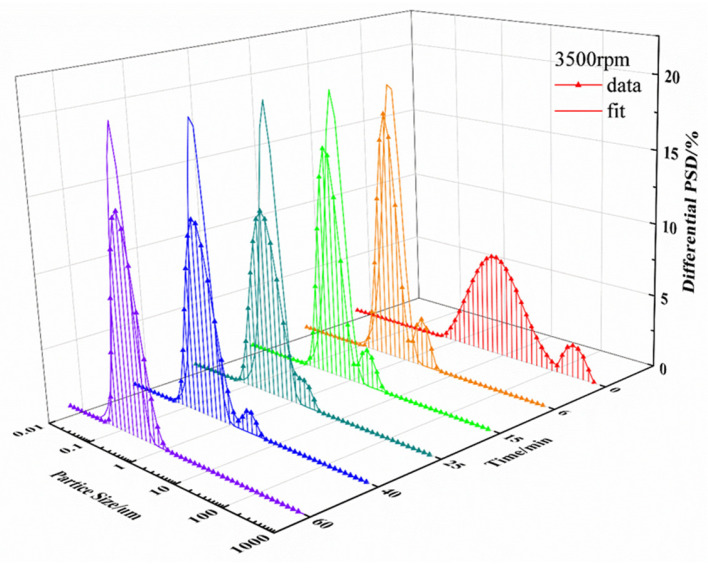
Particle size distribution and fitting curve at grinding speeds of 2000 rpm, 2500 rpm, 3000 rpm, and 3500 rpm.

From the fitting curves for each speed (see Figure 9), it can be seen that the particle size distribution curve during the milling process has a great impact on the accuracy of the model fitting. In general, a normal particle size distribution curve shows a Gaussian distribution. However, the particle size of UA raw materials in suspension shows a bimodal distribution at the beginning of wet grinding, indicating the presence of two kinds of particle sizes in the system. With the increase in milling time, the number of large particles decreases due to grinding, while the proportion of small particles gradually increases. Finally, the overall particle size distribution reaches a unimodal distribution. This phenomenon is more obvious at a grinding speed of 2000 rpm, as reflected in the particle size distribution curve. As grinding time increases, the peak in small particles gradually increases, and the peak in large particles gradually decreases. From Figure 9, the particle size distribution of UA in suspension changes faster from bimodal distribution to unimodal distribution at higher grinding speeds. The comparison of fitting parameters at different grinding speeds in Table 3 shows that the breakage rate constant $K_{1}$ gradually increases with increased grinding speed, indicating that higher grinding efficiency would be obtained for higher grinding speed, and more particles would form during the same milling time.

From Table 3, the variation in breakage frequency constant with different grinding speeds $K_{1}$ shows a similar monotonous trend to *k* in Section 3.1.2. However, the variation in agglomeration frequency constant $K_{2}$ with increasing grinding speeds reaches a minimum value at 2500 rpm.

Then, the agglomeration process was carefully analyzed; in most cases, smaller particles are easier to agglomerate compared to larger particles. The size of daughter particles was impacted by breakage process; the parameters c and p were used to plot the self-similar distribution function $\theta \left(\xi \right)$ under different grinding speeds, whose expression is as follows:

(9) $$\xi =\frac{v}{\epsilon }$$

where ϵ is the volume of parent particle, *v* is the volume of particles generated by parent particle breakage, and ξ is the ratio of them; then, $\theta \left(\xi \right)$ indicates the frequency distribution of different ratios in particle breakage, and the particle breakage distribution $b\left(v|\epsilon \right)$ can be simplified as follows [30]:

(10) $$b\left(v|\epsilon \right)=\frac{p\xi^{c}{\left(1-\xi \right)}^{c+\left(c+1\right)\left(p-2\right)}\Gamma \left(c+\left(c+1\right)\left(p-1\right)+1\right)}{\epsilon \Gamma \left(c+1\right)\Gamma \left(c+\left(c+1\right)\left(p-2\right)+1\right)}$$

(11) $$b\left(v|\epsilon \right)=\frac{\theta \left(\xi \right)}{\epsilon }$$

Based on the value of parameter c, the breakage behavior of particles can be quantitatively described and predicted, thereby providing critical guidance for process optimization. At a situation of c < 0, the particles in the milling system will be worn and broken from colliding with the grinding medium in the grinding chamber to produce fine debris. In actual production, this typically signifies suboptimal process conditions, leading to an overly broad particle size distribution and poor stability, suggesting that process improvements are needed. If c is equal to 0, the particles will be uniformly broken, and the probability of particles of any size during is equal the milling process, suggesting room for improvement in process controllability. When c > 1, the particles with uniform size will be more easily produced in the milling process, representing an efficient and ideal breakage process. This typically corresponds to a narrower particle size distribution and a more stable final product. Therefore, during process development, we can target c > 1 by actively regulating the crushing mechanism through adjustments to stabilizer type/concentration and process parameters (such as selecting appropriate rotational speed) [41].

**Figure 10:**
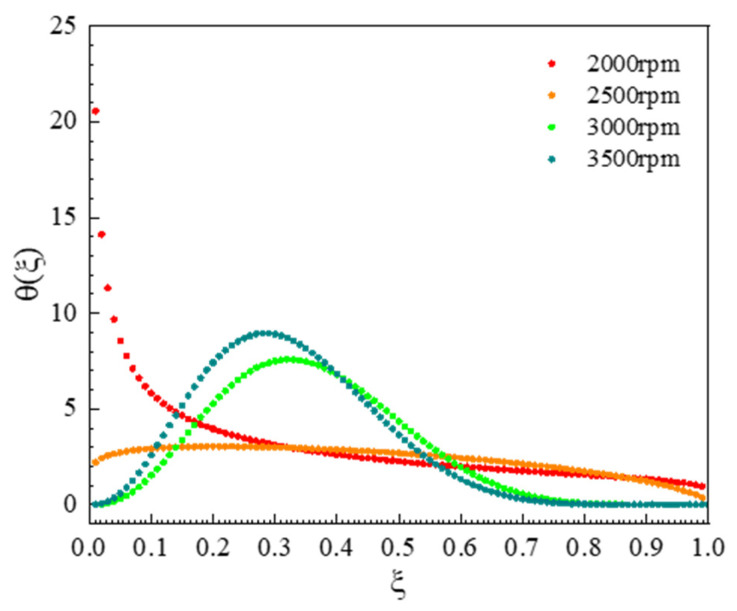
Daughter distribution function at different grinding speeds.

The frequency distribution of daughter particles under different grinding speeds for the UA milling process were shown in Figure 10, and it can be observed that the main breakage behavior of particles is abrasion and erosion caused by the collision of UA particles and grinding beads at a grinding speed 2000 rpm, resulting in a large number of fine particles accounting for less than 10% of the parent particle volume. This is consistent with the gradual reduction in the peak of large particles at 2000 rpm in Figure 9. When grinding speed is improved to 2500 rpm, the breakage behavior of UA particles changes from abrasion milling form to uniform milling form, the particle sizes of daughter particles produced by the breakage of parent particles in the system are different, and the probability for different size daughter particles is basically the same, so that the particle breakage distribution function approaches a straight line. This result coincided with a wider particle size distribution compared to other experimental conditions during the wet milling process for a grinding speed of 2500 rpm, as shown in Figure 9. When the grinding speed is 3000 rpm, the particle breakage distribution function shows a unimodal distribution, the particle size of the daughter particles is concentrated in the range from 20% to 50% of the parent particle volume as shown Figure 10, and the particle size distribution curve is a unimodal distribution. Further increasing the grinding speed to 3500 rpm, the number of daughter particles generated increases, the particle breakage distribution function curve shifts to the left, and the particle size distribution becomes narrower.

## 4. Conclusions

This research carries out wet grinding research on the natural water-insoluble product UA and studies the influence of factors such as the type and concentration of stabilizer, grinding speed, etc., on the UA particle size. Among the seven stabilizers selected for the experiment, Tween-20, as a nonionic surfactant, has a good dispersing effect on the UA suspension in aqueous medium and can effectively inhibit the agglomeration behavior of particles in the system for a period of time (7-day stability inspection), and it is speculated that the agglomeration inhibition of UA in aqueous medium mainly depends on the steric hindrance of stabilizers. Investigating grinding speed in combination with experiments and population balance model simulations revealed that the breakage behavior of UA particles changes due to grinding speed during the milling process; abrasion breakage was predominant at low grinding speeds. As the grinding speed increases, UA particles are more likely to break into a uniform size, and the time taken for the particle size distribution curve to change from an initial bimodal distribution to a unimodal distribution decreases. This research demonstrates that nano-UA suspensions (D50 = 122 nm) can be successfully produced through wet grinding by optimizing the process parameters. This provides theoretical guidance for the preparation of nano-pharmaceuticals.

## Figures and Tables

**Table 1:** Statistical summary of parameter estimation.

Speed/rpm	D*_lim_*/nm	k/nm^(1−n)^ min^−1^	n	R^2^	SSR
2000	292	0.347	2.615	0.987	2.382
2500	241	0.434	2.744	0.988	2.436
3000	245	0.706	2.661	0.985	2.174
3500	213	0.738	2.911	0.988	2.410

**Table 2:** Particle size and scavenging rate of UA grinding products.

Process Time/min	D50/μm	ABS ^1^	Scavenging Rate/%
Blank solution	—	1.508	—
0 min	14.2	1.164	22.81%
10 min	0.183	0.822	45.49%
30 min	0.132	0.556	63.13%
60 min	0.122	0.503	66.64%

^1^ ABS: absorbance value.

**Table 3:** Fitting parameters at different speeds.

Speed/rpm	K1	μ	K2	y1	y2	c	*p*
2000	13.421	0	26.760	0.275	0.273	−0.541	3.464
2500	15.892	0	13.235	0.164	0.137	0.146	2.35
3000	26.701	1	23.900	0.129	0.147	2.807	2.789
3500	35.864	0.042	52.089	0.0112	0.0132	2.646	3.102

## Data Availability

Data is contained within the article.
